# Regional variations in patient selection and procedural characteristics for cryoballoon ablation of atrial fibrillation in the cryo global registry

**DOI:** 10.1007/s10840-023-01582-0

**Published:** 2023-07-28

**Authors:** Fernando Scazzuso, Paweł Ptaszyński, Krzysztof Kaczmarek, K. R. Julian Chun, Surinder Kaur Khelae, Csaba Földesi, Valentine Obidigbo, Kelly A. van Bragt, Young Keun On, Fawzia Al-Kandari, Ken Okumura, On behalf of the Cryo Global Registry Investigators

**Affiliations:** 1 Instituto Cardiovascular de Buenos Aires, Buenos Aires, Argentina; 2 Medical University of Lodz, Łódź, Poland; 3 Cardioangiologisches Centrum Bethanien, Frankfurt, Germany; 4 Institut Jantung Negara, National Heart Institute, Kuala Lumpur, Malaysia; 5 Gottsegen György Országos Kardiovaszkuláris Intézet, Budapest, Hungary; 6 Medtronic, Inc, Mounds View, MN USA; 7 Samsung Medical Center, Sungkyunkwan University School of Medicine, Seoul, Republic of Korea; 8 Chest Disease Hospital, Kuwait, Kuwait; 9 Saiseikai Kumamoto Hospital, Kumamoto, Japan

**Keywords:** Atrial fibrillation, Catheter ablation, Cryoballoon, Registry, Real-world evidence

## Abstract

**Background:**

Cryoballoon ablation is a well-established anatomical approach for pulmonary vein isolation (PVI) in patients with atrial fibrillation (AF). Although widely adopted, regional variations in standards of care have not been well characterized.

**Methods:**

Patients with AF were enrolled in the Cryo Global Registry (NCT02752737) from May 2016 to Sept 2021 at 128 sites in 37 countries and treated with cryoballoon ablation according to local clinical practice. Baseline patient and procedural characteristics were summarized for 8 regions (Central Asia & Russia, East Asia, Europe, Middle East, North America, South Africa, South America, and Southeast Asia). Serious procedure-related adverse events (SAEs) were evaluated in a subset of patients with ≥ 7 days of follow-up.

**Results:**

A total of 3,680 patients undergoing initial PVI for AF were included. Cryoballoon ablation was commonly performed in patients with paroxysmal AF. Mean age ranged from 47 ± 12 years in the Middle East to 64 ± 11 years in East Asia. Mean procedure time was ≤ 95 min in all regions. Average freeze duration ranged from 153 ± 41 s in Southeast Asia to 230 ± 29 s in Central Asia & Russia. Acute procedural success was ≥ 94.7% in all geographies. In 3,126 subjects with ≥ 7 days of follow-up, 122 procedure-related SAEs were reported in 111 patients (3.6%) and remained low in all regions. One procedure-related death was reported during data collection.

**Conclusions:**

Despite regional variations in patient selection and procedural characteristics, PVI using cryoballoon ablation was performed with high acute success and short procedural times around the world.

**Clinical trial registration:**

https://clinicaltrials.gov/ct2/show/NCT02752737

**Graphical Abstract:**

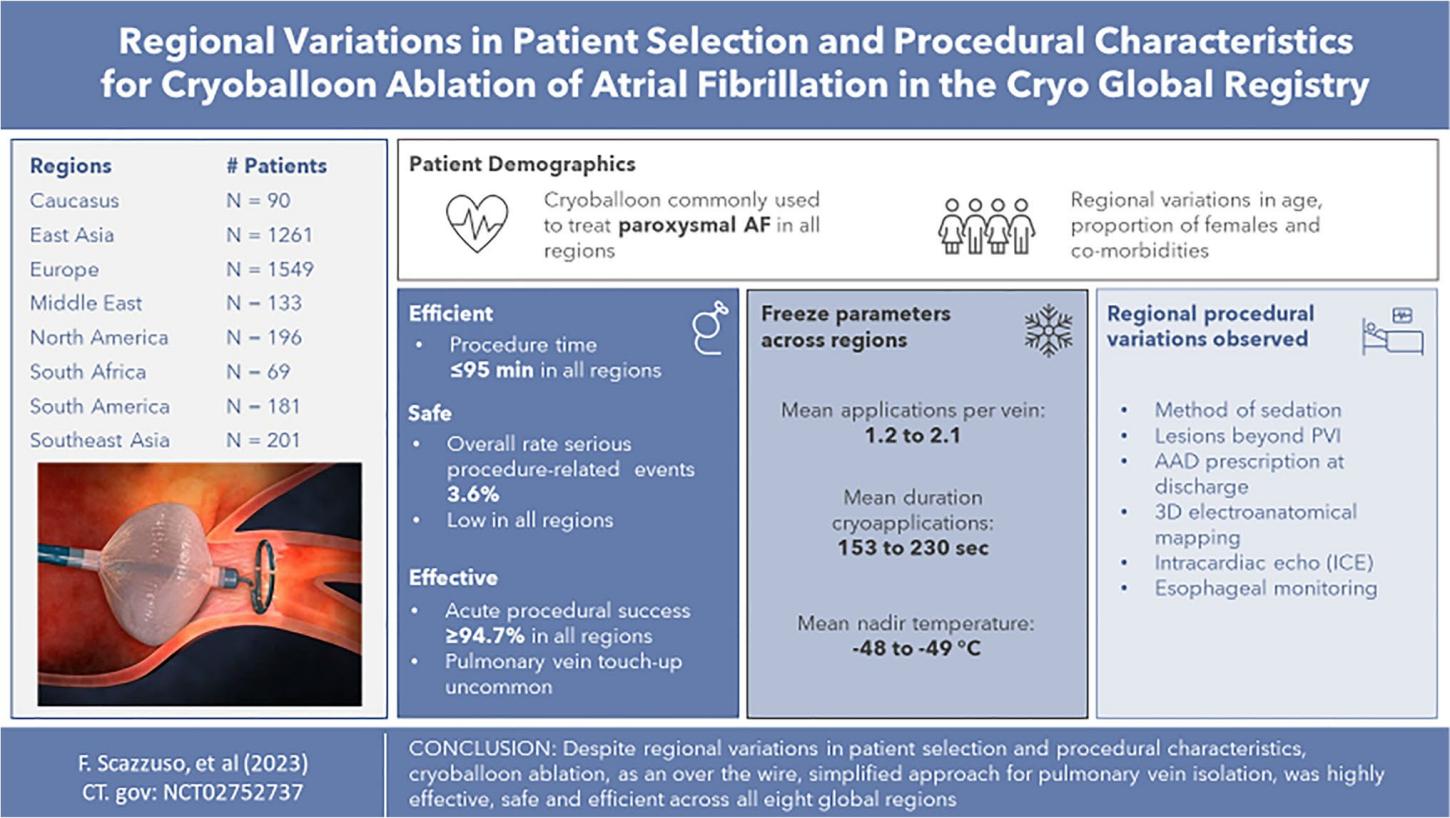

**Supplementary Information:**

The online version contains supplementary material available at 10.1007/s10840-023-01582-0.

## Introduction

Catheter ablation of atrial fibrillation (AF) is a safe and established treatment to prevent atrial arrhythmia recurrences [1]. Historically, radiofrequency ablation (RFA) was considered the gold standard for AF ablation. However, creation of a continuous lesion using a point-by-point approach with RFA can be challenging and requires comprehensive training and expertise [2]. Cryoballoon ablation (CBA) for pulmonary vein isolation (PVI) in patients with AF has been shown to be non-inferior to RFA in terms of efficacy and safety, and CBA has become a well-established alternative treatment option [2–4].

The anatomical approach of the CBA system improved efficiency of PVI by demonstrating shorter and predictable procedures compared to RFA [2, 4, 5] and a short learning curve [6–8]. Yet, despite fairly consistent reports of safety and efficacy with CBA [2, 9, 11], a consolidated reporting of global procedural approaches and dosing have not been described. Also, regional variations in standards-of-care have not been well characterized, and for several global regions data on the CBA procedure is limited. This is the largest sub-analysis of the Cryo Global Registry thus far, with the aim to describe standard-of-care procedure characteristics of CBA in various global regions within this registry.

## Methods

### Study Design

The Cryo Global Registry (NCT02752737) is a global multicenter, prospective, post-market study collecting data of AF ablation procedures conducted with the Arctic Front Advance Family of cryoablation catheters. This registry was sponsored by Medtronic. Data collection adhered to the principles outlined in the Declaration of Helsinki and Good Clinical Practice. Patients provided written informed consent prior to enrollment in the study. Review boards and ethics committees at the participating centers reviewed and approved the study. A global steering committee of physicians advised on data quality, analysis, and publication milestones. All medical procedures were performed according to local standard-of-care. The aim of this sub-analysis of the Cryo Global Registry was to assess regional variations in procedure characteristics of CBA.

## Patient Population

All patients with a planned CBA procedure and a minimum age of 18 years old were eligible for inclusion in the registry. No patients were excluded from participating in the registry based on pre-existing characteristics or medical conditions. In this sub-analysis, data from index CBA procedures were included from patients with paroxysmal AF (PAF; episodes < 7 days) and persistent AF (PsAF; episodes ≥ 7 days and ≤ 12 months) [12] who were enrolled between 02-MAY-2016 and 21-SEP-2021. Exclusion criteria for this sub-analysis included: 1) diagnosis of long-standing persistent AF (continuous AF > 12 months); 2) prior PVI or cavotricuspid isthmus (CTI) ablation; and/or 3) incomplete baseline- and medical history data. Analysis regions were defined as shown in Table 1, and data were evaluated from procedures performed at 128 centers in 37 global countries.

**Table 1:** Analysis regions and countries

Geography	Country Name	Number of Subjects Treated
Central Asia & Russia	Kazakhstan	47
	Russian Federation	43
	**Total**	**90**
East Asia	China	216
	Japan	672
	Korea, South	203
	Taiwan, Republic of China	170
	**Total**	**1261**
	Austria	40
	Belgium	120
	Czech Republic	31
Europe	Germany	470
	Greece	11
	Hungary	126
	Iceland	12
	Italy	161
	Poland	161
	Portugal	30
	Serbia	92
	Slovakia	119
	Spain	17
	Switzerland	22
	United Kingdom	137
	**Total**	**1549**
Middle East	Kuwait	33
	Oman	11
	Saudi Arabia	43
	United Arab Emirates	46
	**Total**	**133**
North America	Mexico	43
	United States of America	153
	**Total**	**196**
South Africa	South Africa	69
	**Total**	**69**
South America	Argentina	160
	Chile	5
	Colombia	16
	**Total**	**181**
	Brunei Darussalam	18
Southeast Asia	Indonesia	1
	Malaysia	122
	Philippines	1
	Singapore	36
	Thailand	23
	**Total**	**201**
Total		**3680**

## Ablation procedure

The CBA procedure using the 23- or 28-mm CBA catheter (Arctic Front Advance or Arctic Front Advance Pro; Medtronic, Inc.) was described in detail previously [13]. In brief, a J-tip guidewire or a dedicated inner-lumen octopolar/decapolar circular mapping catheter (Achieve or Achieve Advance; Medtronic, Inc.) was used to advance the CBA catheter and sheath to each pulmonary vein (PV). Procedures were performed according to the local procedural techniques. Operators independently determined the method used for phrenic nerve monitoring, esophageal temperature monitoring, and procedural imaging. It was recommended by the study protocol that pacing and one other adjunctive method for phrenic nerve monitoring were used during all right-sided PV freeze applications. All freezes were halted upon detection of a decrease in diaphragmatic response. The number of freezes, duration of CBA applications, and need for additional ablation lesions (beyond PVI) were not dictated by the protocol, but the operators were required to confirm PVI by entrance and/or exit block. Patients were managed with periprocedural anticoagulation and antiarrhythmic drug (AAD) prescription per the operator’s directions, and patient discharge occurred according to the center’s standard-of-care method.

## Study Endpoints

The safety endpoint included all serious device- and/or procedure-related adverse events (AEs) evaluated in a subset of patients with ≥ 7 days of follow-up. Relatedness to the procedure and seriousness of events (*e.g.,* leading to death or a serious deterioration of health) were classified by each operator. Arrhythmias classified by the physician as a procedure-related serious adverse event were excluded from the primary safety endpoint if arrhythmia onset occurred post-discharge. The primary efficacy endpoint was acute procedural success for PVI, defined as attempted electrical isolation of all major PVs or their anomalous equivalents with CBA. Ancillary objectives were non-serious device- and procedure-related events, total procedure time, total fluoroscopy time, mean duration of cryoablation, incidence of CTI ablation, and amount of ablation outside of PV and CTI locations. A composite of serious device- and/or procedure-related AEs of interest were analyzed Ad Hoc.

## Statistical Analysis

Continuous variables were summarized as mean and standard deviation with median and inter-quartile range (IQR). Categorical variables were summarized as number of subjects and percentages. Baseline and procedural characteristics were compared between eight global regions. Acute procedural success rate by subjects were calculated as the number of subjects with acute procedural success divided by the number of subjects with an attempted CBA procedure. Statistical analyses were performed with SAS software version 9.4 (SAS Institute, Cary, North Carolina).

## Results

### Patient Baseline Characteristics

A total of 3,680 patients were analyzed in this sub-study of the Cryo Global Registry; patient baseline characteristics per region are summarized in Table 2. Cryoablation procedures were more commonly performed in males in all regions except Central Asia & Russia. The mean age ranged from 47 ± 12 years in the Middle East to 64 ± 11 years in East Asia. Most procedures were performed in patients with PAF in all regions. The largest proportion of cases performed in PsAF patients was observed in South Africa (42.0%). Median time from AF diagnosis to AF catheter ablation ranged from 0.4 (0—2) years in the Middle East to 2.0 (1—5) years in Central Asia & Russia. The percentage of AF patients with a history of atrial flutter ranged from 2.2% in Central Asia & Russia to 15.3% in North America.

**Table 2:** Subject characteristics

Subject Characteristics	Central Asia & Russia (*N* = 90)	East Asia (*N* = 1261)	Europe (*N* = 1549)	Middle East (*N* = 133)	North America (*N* = 196)	South Africa (*N* = 69)	South America (*N* = 181)	Southeast Asia (*N* = 201)
Sex (Male)	41 (45.6%)	834 (66.1%)	960 (62.0%)	94 (70.7%)	129 (65.8%)	53 (76.8%)	122 (67.4%)	110 (54.7%)
Age (Years)	60 ± 9	64 ± 11	62 ± 11	47 ± 12	62 ± 11	60 ± 12	56 ± 12	58 ± 11
BMI (kg/m2)	29 ± 5	25 ± 4	28 ± 5	30 ± 7	30 ± 6	30 ± 6	27 ± 5	27 ± 6
CHA_2_DS_2_-VASc Score	2.6 ± 1.2	2.2 ± 1.5	2.1 ± 1.5	1.0 ± 1.1	1.8 ± 1.3	1.6 ± 1.5	1.1 ± 1.0	2.2 ± 1.4
Paroxysmal AF	74 (82.2%)	868 (68.8%)	1104 (71.3%)	125 (94.0%)	190 (96.9%)	40 (58.0%)	156 (86.2%)	180 (89.6%)
Persistent AF	16 (17.8%)	393 (31.2%)	445 (28.7%)	8 (6.0%)	6 (3.1%)	29 (42.0%)	25 (13.8%)	21 (10.4%)
Time Since First AF Diagnosis (Years)	2.0 (1–5)	0.5 (0–2)	1.8 (1–5)	0.4 (0–2)	1.5 (0–4)	0.9 (0–3)	1.5 (1–3)	1.2 (0–3)
LA Diameter (mm)	40 ± 6	40 ± 6	43 ± 8	38 ± 6	40 ± 7	43 ± 9	40 ± 6	38 ± 7
Ventricular EF (%)	61 ± 7	61 ± 9	58 ± 9	58 ± 6	60 ± 9	59 ± 8	61 ± 6	61 ± 10
Number of Previously Failed AADs	1.0 (1–2)	0.0 (0–1)	1.0 (0–1)	0.0 (0–0)	1.0 (0–1)	1.0 (0–1)	1.0 (1–1)	0.0 (0–0)
Hypertension	69 (76.7%)	657 (52.1%)	948 (61.2%)	33 (24.8%)	124 (63.3%)	25 (36.2%)	57 (31.5%)	117 (58.2%)
Heart Failure	71 (78.9%)	475 (37.7%)	550 (35.5%)	41 (30.8%)	41 (20.9%)	20 (29.0%)	15 (8.3%)	80 (39.8%)
Prior MI	4 (4.4%)	24 (1.9%)	50 (3.2%)	2 (1.5%)	10 (5.1%)	0 (0.0%)	2 (1.1%)	5 (2.5%)
Prior Stroke/TIA	4 (4.4%)	96 (7.6%)	85 (5.5%)	4 (3.0%)	10 (5.1%)	2 (2.9%)	5 (2.8%)	15 (7.5%)
History of Coronary Artery Disease	16 (17.8%)	118 (9.4%)	164 (10.6%)	10 (7.5%)	42 (21.4%)	3 (4.3%)	5 (2.8%)	32 (15.9%)
Diabetes	6 (6.7%)	183 (14.5%)	191 (12.3%)	22 (16.5%)	25 (12.8%)	9 (13.0%)	9 (5.0%)	54 (26.9%)
Sleep Apnea	0 (0.0%)	36 (2.9%)	76 (4.9%)	7 (5.3%)	37 (18.9%)	2 (2.9%)	10 (5.5%)	4 (2.0%)

Data are N(%), mean ± SD or median (IQR); AF, atrial fibrillation; LA, left atrium; EF, ejection fraction; AAD, antiarrhythmic drug; BMI, body mass index; MI, myocardial infarction; TIA, transient ischemic attack

Large variations in the proportion of patients with comorbidities at baseline were observed, as recorded during the index procedure. The percentage of patients with heart failure undergoing CBA ranged from 8.3% in South Africa to 78.9% in Central Asia & Russia. Presence of hypertension ranged from 24.8% in the Middle East to 76.2% in Central Asia & Russia. Co-existence of diabetes in patients undergoing CBA for AF was low in South America (5.0%) and Central Asia & Russia (6.7%), with the highest percentage of diabetes reported in Southeast Asia (26.9%). A history of coronary artery disease and the presence of sleep apnea were most often reported in North American patients (21.4% and 18.9%, respectively).

## Procedural Characteristics and Acute Efficacy

Procedural parameters per region can be found in Table 3. Median procedure time ranged from 61 (49–68) min in Central Asia & Russia to 95 (76–120) min in Southeast Asia, and median fluoroscopy time was below 20 min across all regions. Fluoroscopy was the most frequent intraprocedural visualization method in all regions; however, in North America, intracardiac echocardiography (ICE; 86.3%) and 3D electroanatomical mapping (63.3%) were commonly utilized in addition to fluoroscopy. The sedation method varied per region. General anesthesia was administered in almost all CBA cases in South Africa (98.6%) and South America (99.4%), while conscious sedation was the most prevalent sedation method in Europe (76.8%). Large variations in the use of esophageal monitoring were observed ranging from 0% in South Africa to 87.3% in South America. Phrenic nerve monitoring during right-sided CBA was close to 100% in all regions, and the use of compound motor action potential (CMAP) for phrenic nerve monitoring was highest in Central Asia & Russia (54.4%), East Asia (50.0%), and Southeast Asia (48.3%). Same-day discharge was uncommon in all geographies except for North and South America (37.8% and 26.5%), respectively. AADs were regularly prescribed in all regions for the management of atrial arrhythmias after the procedure, with the highest prescription rates at discharge in Central Asia & Russia (85.6%) and North America (72.1%).

**Table 3:** Procedural characteristics

Procedure Characteristics	Central Asia & Russia (*N* = 90)	East Asia (*N* = 1261)	Europe (*N* = 1549)	Middle East (*N* = 133)	North America (*N* = 196)	South Africa (*N* = 69)	South America (*N* = 181)	Southeast Asia (*N* = 201)
Total Lab Occupancy Time (Min)	91 (80–109)	125 (96–167)	125 (100–155)	147 (120–180)	166 (147–197)	169 (145–195)	100 (85–125)	165 (140–200)
Total Procedure Time (Min)	61 (49–68)	71 (57–95)	75 (57–100)	80 (62–105)	89 (71–107)	82 (60–96)	65 (55–81)	95 (76–120)
Left Atrial Dwell Time (Min)	36 (28–48)	47 (36–63)	50 (36–67)	55 (40–70)	62 (49–77)	53 (41–67)	42 (33–55)	61 (50–82)
Total Fluoroscopy Time (Min)	8 (6–15)	16 (10–33)	11 (7–17)	19 (12–34)	9 (6–13)	13 (9–20)	10 (7–14)	19 (14–30)
Sedation Method								
- General Anesthesia	48 (53.3%)	495 (39.3%)	354 (22.9%)	49 (36.8%)	158 (80.6%)	68 (98.6%)	180 (99.4%)	119 (59.2%)
—Conscious Sedation	42 (46.7%)	755 (59.9%)	1190 (76.8%)	84 (63.2%)	38 (19.4%)	1 (1.4%)	1 (0.6%)	81 (40.3%)
Mapping/Navigation Tools								
- Fluoroscopy	89 (98.9%)	1127 (89.4%)	1396 (90.1%)	127 (95.5%)	182 (92.9%)	67 (97.1%)	177 (97.8%)	155 (77.1%)
- ICE	50 (55.6%)	445 (35.3%)	246 (15.9%)	5 (3.8%)	166 (84.7%)	0 (0.0%)	19 (10.5%)	44 (21.9%)
—3D Mapping	0 (0.0%)	483 (38.3%)	86 (5.6%)	1 (0.8%)	124 (63.3%)	1 (1.4%)	0 (0.0%)	41 (20.4%)
Esophageal Monitoring	27 (30.0%)	789 (62.6%)	541 (34.9%)	12 (9.0%)	154 (78.6%)	0 (0.0%)	158 (87.3%)	14 (7.0%)
Phrenic Nerve Monitoring	90 (100.0%)	1254 (99.4%)	1527 (98.6%)	130 (97.7%)	196 (100.0%)	69 (100.0%)	179 (98.9%)	201 (100.0%)
Discharge Same-Day	1 (1.1%)	4 (0.3%)	64 (4.1%)	8 (6.0%)	74 (37.8%)	0 (0.0%)	48 (26.5%)	1 (0.5%)
AAD at Discharge	77 (85.6%)	381 (52.9%)	622 (45.8%)	77 (63.6%)	71 (55.0%)	29 (54.7%)	131 (90.3%)	59 (36.4%)

Data are N(%) or median (IQR); ICE, Intracardiac Echocardiography; AAD, antiarrhythmic drug

Acute success and ablation parameters are further specified in Table 4. Acute procedural success was ≥ 94.7% in all regions. The number of freeze applications per vein ranged from 1.2 ± 0.4 in Central Asia & Russia (median (IQR): 1 (1, 1)) to 2.1 ± 1.0 in North America (median (IQR): 2 (2, 2)). Average duration of CBA applications was 230 ± 29 s in Central Asia & Russia (median (IQR): 240 (240, 240)) compared to 156 ± 46 s in North America (median (IQR): 180 (120, 180)). Added focal ablation “touch-up” for acute PVI were required in a minority of cases across all geographies.

**Table 4:** Ablation parameters and acute success

Ablation Characteristics	Central Asia & Russia (*N* = 90)	East Asia (*N* = 1261)	Europe (*N* = 1549)	Middle East (*N* = 133)	North America (*N* = 196)	South Africa (*N* = 69)	South America (*N* = 181)	Southeast Asia (*N* = 201)
PVI Acute Procedural Success	90(100.0%)	1194 (94.7%)	1471 (95.0%)	129 (97.0%)	191 (97.4%)	68 (98.6%)	172 (95.0%)	196 (97.5%)
PVI Focal RF Touch-up	0 (0.0%)	60 (4.8%)	95 (6.1%)	1 (0.8%)	9 (4.6%)	9 (13.0%)	15 (8.3%)	11 (5.5%)
PVI Focal Cryo Touch-up	0 (0.0%)	7 (0.6%)	0 (0.0%)	0 (0.0%)	0 (0.0%)	0 (0.0%)	0 (0.0%)	0 (0.0%)
Number of Applications Per Vein								
- Mean	1.2 ± 0.4	1.6 ± 0.9	1.5 ± 1.0	1.5 ± 0.8	2.1 ± 1.0	1.6 ± 0.7	1.3 ± 0.5	2.0 ± 1.0
- Median	1 (1, 1)	1 (1, 2)	1 (1, 2)	1 (1, 2)	2 (2, 2)	1 (1, 2)	1 (1, 1)	2 (1, 2)
Duration of Cryoapplication (Sec)								
- Mean	230 ± 29	158 ± 44	197 ± 53	211 ± 60	156 ± 46	225 ± 51	210 ± 40	153 ± 41
- Median	240 (240, 240)	180 (120, 180)	180 (180, 240)	240 (180, 240)	180 (120, 180)	240 (240, 240)	240 (180, 240)	180 (120, 180)
Cryoballoon Nadir Temperature (°C)	-49 ± 7	-49 ± 7	-48 ± 7	-47 ± 7	-49 ± 8	-48 ± 8	-48 ± 6	-49 ± 6
CTI-ablations	1 (1.1%)	356 (28.2%)	29 (1.9%)	7 (5.3%)	40 (20.4%)	10 (14.5%)	15 (8.3%)	19 (9.5%)
Non-PVI, Non-CTI ablations	47 (52.2%)	206 (16.3%)	10 (0.6%)	2 (1.5%)	21 (10.7%)	3 (4.3%)	16 (8.8%)	9 (4.5%)
- LA AF Trigger	47 (52.2%)	48 (3.8%)	5 (0.3%)	1 (0.8%)	4 (2.0%)	0 (0.0%)	16 (8.8%)	4 (2.0%)
- RA AF Trigger	36 (40.0%)	9 (0.7%)	2 (0.1%)	0 (0.0%)	2 (1.0%)	0 (0.0%)	0 (0.0%)	0 (0.0%)
- SVC Trigger	0 (0.0%)	30 (2.4%)	0 (0.0%)	0 (0.0%)	0 (0.0%)	1 (1.4%)	0 (0.0%)	1 (0.5%)
- Mitral Valve Isthmus or Line	0 (0.0%)	6 (0.5%)	0 (0.0%)	0 (0.0%)	0 (0.0%)	1 (1.4%)	0 (0.0%)	0 (0.0%)
- Left sided roofline	0 (0.0%)	106 (8.4%)	1 (0.1%)	1 (0.8%)	2 (1.0%)	0 (0.0%)	0 (0.0%)	3 (1.5%)
- CFAE	0 (0.0%)	5 (0.4%)	0 (0.0%)	0 (0.0%)	5 (2.6%)	0 (0.0%)	0 (0.0%)	0 (0.0%)
- Other	0 (0.0%)	48 (3.8%)	3 (0.2%)	0 (0.0%)	10 (5.1%)	1 (1.4%)	0 (0.0%)	2 (1.0%)

Data are N(%), mean ± SD or median (IQR); PVI, pulmonary vein isolation; RF, Radiofrequency; CTI: cavotricuspid isthmus; LA, left atrial; RA, right atrial; AF, atrial fibrillation; SVC, superior vena cava; CFAE, complex fractionated atrial electrograms

Other ablations beyond PVI varied widely per region. The percentage of patients receiving CTI ablations was > 20% in East Asia (28.2%) and North America (20.4%). In East Asia, 16.3% received additional non-PVI/non-CTI ablations, of which half were left-sided roofline lesions (8.4%). In Central Asia & Russia, the percentage of CTI ablations was low (1.1%); however, more than half of patients received additional non-PVI/non-CTI ablations, all being left and right atrial trigger ablations (52.2% and 40.0%, respectively). Additional ablations were relatively uncommon in Europe (*i.e.,* 1.9% CTI and 0.6% non-PVI/non-CTI) and the Middle East (*i.e.,* 5.3% CTI and 1.5% non-PVI/non-CTI).

## Procedural Safety

A subset of 3,126 out of 3,680 patients had ≥ 7 days of follow-up at the time of the analysis, and a total of 122 serious device- and/or procedure-related AEs were reported in 111 patients (3.6%; full listing in Supplement Table 1). In addition, 50 device- and/or procedure-related AEs were reported as non-serious in 46 patients (1.5%). Cardiac tamponade/perforation/pericardial effusion and stroke/transient ischemic attacks were uncommon in all regions (Table 5). A total of 40 phrenic nerve paralyses were reported as adverse events in 3,126 patients (1.3%), of which 15 were classified by the physician as serious. Of the 40 total phrenic nerve paralyses, 10 were resolved at the time of patient discharge from the hospital. No atrioesophageal fistula or PV stenosis were reported in any region. One death occurred 24 days post-procedure in North America as a consequence of a cerebrovascular accident that occurred immediately after the procedure.

**Table 5:** Device- and/or procedure-related adverse events

Adverse Events	Central Asia & Russia (*N* = 90)	East Asia (*N* = 907)	Europe (*N* = 1423)	Middle East (*N* = 129)	North America (*N* = 168)	South Africa (*N* = 60)	South America (*N* = 176)	Southeast Asia (*N* = 173)
Total Serious Device- and/or Procedure-Related AE	2 (2, 2.2)	22 (21, 2.3)	72 (65, 4.6)	0 (0, 0.0)	16 (13, 7.7)	1 (1, 1.7)	4 (4, 2.3)	5 (5, 2.9)
Total Non-Serious Device- and/or Procedure-Related AE	2 (2, 2.2)	11 (10, 1.1)	25 (22, 1.5)	0 (0, 0.0)	4 (4, 2.4)	0 (0, 0.0)	7 (7, 4)	1 (1, 0.6)
Composite of Serious Device- and/or Procedure- Related AEs of Interest	1 (1,1.1)	12 (12, 1.3)	38 (36, 2.5)	0 (0, 0.0)	5 (4, 2.4)	1 (1, 1.7)	3 (3, 1.7)	4 (4, 2.3)
-Cardiac Tamponade, Perforation, Pericardial Effusion	0 (0, 0.0)	2 (2, 0.2)	8 (8, 0.6)	0 (0, 0.0)	2 (2, 1.2)	0 (0, 0.0)	0 (0, 0.0)	1 (1, 0.6)
-Groin-site Complication	0 (0, 0.0)	5 (5, 0.6)	15 (13, 0.9)	0 (0, 0.0)	2 (2, 1.2)	1 (1, 1.7)	2 (2, 1.1)	1 (1, 0.6)
-MI or Ischemic Cardiac Event	0 (0, 0.0)	0 (0, 0.0)	3 (3, 0.2)	0 (0, 0.0)	0 (0, 0.0)	0 (0, 0.0)	0 (0, 0.0)	1 (1, 0.6)
-Phrenic Nerve Paralysis	0 (0, 0.0)	4 (4, 0.4)	10 (10, 0.7)	0 (0, 0.0)	0 (0, 0.0)	0 (0, 0.0)	1 (1, 0.6)	0 (0, 0.0)
-Stroke or TIA of any Cause	1 (1, 1.1)	1 (1, 0.1)	2 (2, 0.1)	0 (0, 0.0)	1 (1, 0.6)	0 (0, 0.0)	0 (0, 0.0)	1 (1, 0.6)

Data are N Events (N Subjects, % Subjects); SAE: Serious adverse event; AE, adverse event; MI, myocardial infarction; TIA, transient ischemic attack

## Discussion

CBA was commonly used to treat PAF patients in all regions. Regional variations were observed in baseline characteristics including mean age, prevalence of comorbidities, and proportion of female and PsAF patients undergoing CBA. Procedures were efficient overall, with the longest average procedure duration of 95 min observed in Southeast Asia. Acute procedural success was ≥ 94.7% in all regions, and the need for focal touch-up was uncommon. The median duration of freeze applications was 180–240 s across all regions. Regional procedural differences were observed in the method of sedation, lesions beyond PVI, and AAD prescription at discharge. In addition, the use of 3D electroanatomical mapping, ICE, and esophageal monitoring varied significantly across regions. Despite variations in patients (demographic and baseline characteristics) and procedure characteristics; total serious device- and/or procedure-related AEs were reported in 3.6% of patients and remained low in all global regions.

The standard-of-care method of catheter ablation for AF was first described by Cappato et al*.* by means of a worldwide survey in a questionnaire format that was published in 2005 [14]. Since then, modern registries were conducted and are ongoing. These registries have proven useful to describe the patient population receiving catheter ablation treatment, routine clinical practice methods, and clinical outcome in a real-world evidence setting [15–17]. This sub-analysis of the Cryo Global Registry aims to describe regional variations and similarities in CBA procedures using the Arctic Front Advance family of catheters in patients undergoing PVI for the treatment of AF.

The cryoballoon was designed to achieve PVI with a single shot of (cryothermal) energy. Several studies have shown that the anatomical approach of CBA results in increased procedural efficiency compared to RFA, demonstrating shorter, more predictable procedures that are safe and effective across operators [2, 4, 5, 7, 8, 11]. The present analysis of the Cryo Global Registry supports these earlier findings in a global, real-world evidence setting. Regional procedure and fluoroscopy times in the Cryo Global Registry were comparable, or shorter, compared to CBA procedures in other recent registries [4, 11, 18–20]. The use of additional imaging/navigational tools (*e.g.,* ICE and 3D electroanatomical mapping) varied per region in the present study. Depending on the country and local reimbursement, the usage of additional imaging tools will likely add costs to the CBA procedure, which may influence the operator’s decision to use them. With CBA being an over the wire simplified procedure; the observed differences in procedural techniques did not largely affect regional outcomes, and acute efficacy and safety rates are in alignment with earlier findings [2, 9–11].

Advancements in cryoballoon technology have led to changes in dosing strategies for CBA. The Arctic Front Advance cryoballoon achieved similar temperature profiles to the earlier Arctic Front cryoballoon, but at a faster rate, allowing for a shortening of freeze duration [21]. Contemporary dosing strategies (*e.g.,* single-freeze applications of 180–240 s, shorter 120 s double-freeze applications, or time-to-isolation guided ablation) have significantly shortened procedural and fluoroscopy times, with similar rates of safety and efficacy [3, 22–26]. These trends can also be observed in the current registry analysis. In most regions, except North America and Southeast Asia, operators applied a median of one freeze application per vein with a median of 180–240 s freeze duration. North America and Southeast Asia reported a median freeze application of two per vein with a median duration of 180 s (mean 156 and 153 s, respectively). However, the Cryo Global Registry did not collect information on the reason for a second application, nor was the (visualization of) time-to-isolation collected. This dataset is therefore limited in further describing the dosing protocol regarding bonus freezes or time-to-isolation guided freeze durations. Consequently, more studies are needed to better understand the effects of dosing strategy on acute safety and efficacy in a real world setting.

## Study Limitations

This sub-analysis of the Cryo Global Registry describes standard-of-care procedure characteristics, acute efficacy, and safety across different global regions. A variable number of centers and enrollments happened across regions, which could introduce bias in the regional differences observed. Procedural guidance, phrenic nerve and esophageal temperature monitoring methods, and AAD prescriptions post-ablation were not prescribed by the study protocol. It should also be noted that self-reporting of adverse events could lead to underreporting of events, and the minimum follow-up time of 7 days for adverse events in this sub-analysis is likely to be insufficient time to capture atrio-esophageal fistula or pulmonary vein stenosis events. In addition, not all geographical regions started enrollment at the same time during the enrollment period between May 2016 to October 2021, and temporal trends in procedural methods (*e.g.,* same day discharge, first-line ablation, or dosing strategies) could have impacted regions differently. And lastly, cryoballoon generation use is likely influenced by timing of site activation in the Registry and the availability and cost of AFA Pro in each region. Procedural cost and affordability issues in some global regions could cause bias in patient availability and selection for the CBA procedure, which ultimately results in potential socioeconomic bias. Nevertheless, this underlines even more so the importance of characterizing real-world CBA procedures in different socioeconomic regions.

## Conclusion

Despite regional variations in patient selection and procedural characteristics, CBA had a high acute procedural success rate (≥ 95%) across all regions. Procedures were efficient with a median procedure duration ≤ 95 min and serious device- and/or procedure-related adverse events (3.6%) were uncommon.

### Supplementary Information

Below is the link to the electronic supplementary material.

Supplementary file1 (DOCX 23 KB)

## Abbreviations

AAD: Antiarrhythmic Drugs
AE: Adverse Event
AF: Atrial Fibrillation
CBA: Cryoballoon Ablation
CTI: Cavotricuspid Isthmus
PAF: Paroxysmal Atrial Fibrillation
PsAF: Persistent Atrial Fibrillation
PVI: Pulmonary Vein Isolation
RFA: Radiofrequency Ablation
