# Supplementary material for: Regional variations in patient selection and procedural characteristics for cryoballoon ablation of atrial fibrillation in the cryo global registry
Source: J Interv Card Electrophysiol. 2023 Jul 28;67(3):493–501. doi: 10.1007/s10840-023-01582-0 (PMC11016010; doi:10.1007/s10840-023-01582-0)
Supplement: Supplementary file 1 — Supplementary file1 (DOCX 23 KB) [file 10840_2023_1582_MOESM1_ESM.docx]

**Supplement**

**Supplement Table 1: Specification of all Serious Procedure-related Adverse Events**

| **Serious Procedure Related Adverse Event** | **Caucasus (N = 90)** | **East Asia (N = 907)** | **Europe (N = 1423)** | **Middle East (N = 129)** | **North America (N = 168)** | **South Africa (N = 60)** | **South America (N = 176)** | **Southeast Asia (N = 173)** |
| --- | --- | --- | --- | --- | --- | --- | --- | --- |
| **Total** | **2 (2, 2.2)** | **22 (21, 2.3)** | **72 (65, 4.6)** | **0 (0, 0.0)** | **16 (13, 7.7)** | **1 (1, 1.7)** | **4 (4, 2.3)** | **5 (5, 2.9)** |
| Altered state of consciousness | 0 (0, 0.0) | 1 (1, 0.1) | 0 (0, 0.0) | 0 (0, 0.0) | 0 (0, 0.0) | 0 (0, 0.0) | 0 (0, 0.0) | 0 (0, 0.0) |
| Atrial septal defect | 0 (0, 0.0) | 0 (0, 0.0) | 1 (1, 0.1) | 0 (0, 0.0) | 0 (0, 0.0) | 0 (0, 0.0) | 0 (0, 0.0) | 0 (0, 0.0) |
| Atrioventricular block complete | 0 (0, 0.0) | 1 (1, 0.1) | 0 (0, 0.0) | 0 (0, 0.0) | 0 (0, 0.0) | 0 (0, 0.0) | 0 (0, 0.0) | 0 (0, 0.0) |
| Cardiac failure | 0 (0, 0.0) | 0 (0, 0.0) | 1 (1, 0.1) | 0 (0, 0.0) | 1 (1, 0.6) | 0 (0, 0.0) | 0 (0, 0.0) | 0 (0, 0.0) |
| Cardiac tamponade, perforation, Pericardial effusion | 0 (0, 0.0) | 2 (2, 0.2) | 8 (8, 0.6) | 0 (0, 0.0) | 2 (2, 1.2) | 0 (0, 0.0) | 0 (0, 0.0) | 1 (1, 0.6) |
| Face injury | 0 (0, 0.0) | 0 (0, 0.0) | 0 (0, 0.0) | 0 (0, 0.0) | 0 (0, 0.0) | 0 (0, 0.0) | 1 (1, 0.6) | 0 (0, 0.0) |
| Femoral artery aneurysm | 0 (0, 0.0) | 0 (0, 0.0) | 1 (1, 0.1) | 0 (0, 0.0) | 0 (0, 0.0) | 0 (0, 0.0) | 0 (0, 0.0) | 0 (0, 0.0) |
| Fluid overload | 0 (0, 0.0) | 0 (0, 0.0) | 0 (0, 0.0) | 0 (0, 0.0) | 1 (1, 0.6) | 0 (0, 0.0) | 0 (0, 0.0) | 0 (0, 0.0) |
| Groin-site complication | 0 (0, 0.0) | 5 (5, 0.6) | 15 (13, 0.9) | 0 (0, 0.0) | 2 (2, 1.2) | 1 (1, 1.7) | 2 (2, 1.1) | 1 (1, 0.6) |
| Headache | 0 (0, 0.0) | 0 (0, 0.0) | 0 (0, 0.0) | 0 (0, 0.0) | 1 (1, 0.6) | 0 (0, 0.0) | 0 (0, 0.0) | 0 (0, 0.0) |
| Lip injury | 0 (0, 0.0) | 0 (0, 0.0) | 0 (0, 0.0) | 0 (0, 0.0) | 1 (1, 0.6) | 0 (0, 0.0) | 0 (0, 0.0) | 0 (0, 0.0) |
| Myocardial infarction or ischemic cardiac event | 0 (0, 0.0) | 0 (0, 0.0) | 3 (3, 0.2) | 0 (0, 0.0) | 0 (0, 0.0) | 0 (0, 0.0) | 0 (0, 0.0) | 1 (1, 0.6) |
| Pericarditis | 0 (0, 0.0) | 0 (0, 0.0) | 1 (1, 0.1) | 0 (0, 0.0) | 3 (3, 1.8) | 0 (0, 0.0) | 0 (0, 0.0) | 0 (0, 0.0) |
| Phrenic nerve paralysis | 0 (0, 0.0) | 4 (4, 0.4) | 10 (10, 0.7) | 0 (0, 0.0) | 0 (0, 0.0) | 0 (0, 0.0) | 1 (1, 0.6) | 0 (0, 0.0) |
| Postoperative hypotension | 0 (0, 0.0) | 1 (1, 0.1) | 1 (1, 0.1) | 0 (0, 0.0) | 2 (2, 1.2) | 0 (0, 0.0) | 0 (0, 0.0) | 0 (0, 0.0) |
| Presyncope | 0 (0, 0.0) | 0 (0, 0.0) | 1 (1, 0.1) | 0 (0, 0.0) | 0 (0, 0.0) | 0 (0, 0.0) | 0 (0, 0.0) | 1 (1, 0.6) |
| Pulmonary or bronchial complication | 0 (0, 0.0) | 1 (1, 0.1) | 8 (8, 0.6) | 0 (0, 0.0) | 0 (0, 0.0) | 0 (0, 0.0) | 0 (0, 0.0) | 0 (0, 0.0) |
| Puncture site discharge | 0 (0, 0.0) | 1 (1, 0.1) | 0 (0, 0.0) | 0 (0, 0.0) | 0 (0, 0.0) | 0 (0, 0.0) | 0 (0, 0.0) | 0 (0, 0.0) |
| Pyrexia | 0 (0, 0.0) | 2 (2, 0.2) | 0 (0, 0.0) | 0 (0, 0.0) | 0 (0, 0.0) | 0 (0, 0.0) | 0 (0, 0.0) | 0 (0, 0.0) |
| Sepsis | 0 (0, 0.0) | 0 (0, 0.0) | 1 (1, 0.1) | 0 (0, 0.0) | 0 (0, 0.0) | 0 (0, 0.0) | 0 (0, 0.0) | 0 (0, 0.0) |
| Stress cardiomyopathy | 0 (0, 0.0) | 0 (0, 0.0) | 0 (0, 0.0) | 0 (0, 0.0) | 1 (1, 0.6) | 0 (0, 0.0) | 0 (0, 0.0) | 0 (0, 0.0) |
| Stroke or TIA of any cause | 1 (1, 1.1) | 1 (1, 0.1) | 2 (2, 0.1) | 0 (0, 0.0) | 1 (1, 0.6) | 0 (0, 0.0) | 0 (0, 0.0) | 1 (1, 0.6) |
| Supraventricular arrhythmias | 1 (1, 1.1) | 3 (3, 0.3) | 18 (18, 1.3) | 0 (0, 0.0) | 1 (1, 0.6) | 0 (0, 0.0) | 0 (0, 0.0) | 0 (0, 0.0) |
| Urinary retention | 0 (0, 0.0) | 0 (0, 0.0) | 1 (1, 0.1) | 0 (0, 0.0) | 0 (0, 0.0) | 0 (0, 0.0) | 0 (0, 0.0) | 0 (0, 0.0) |
